# Supplementary material for: Robust and adjustable dynamic scattering compensation for high-precision deep tissue optogenetics
Source: Commun Biol. 2023 Jan 31;6:128. doi: 10.1038/s42003-023-04487-w (PMC9889738; doi:10.1038/s42003-023-04487-w)
Supplement: Supplementary file 2 — Supplementary Information [file 42003_2023_4487_MOESM2_ESM.pdf]

Supplementary Information

Supplementary Method 1. System daily alignment procedure before the experiments

Before the operation each day, the system should be tuned to maintain optimal performance of the fCOAT system by ensuring fine alignment between the focusing objective, imaging objectives and the collecting devices.

1. **Incident beam normal to FLC-SLM.** The FLC-SLM used in the experiments required normal incident light and each pixel of it acted as a half-wave plate, thus the polarization state would be changed after the reflection. We reduced the diaphragm diameter and tilted the SLM to achieve the requirement above by observing the playback beam coincident with the incident light on the diaphragm to maximize the modulation efficiency measured by the dynamometer.
2. **Digitally correct for the tilt and shift misalignment in the focusing module.** We loaded an astigmatic 'X' image (Fig. S2) on the SLM as the alignment basis. We used a 2D translation stage to fine-tuning the position of the SLM on the plane that normal to the incident light. The loaded image had a clear center point and marginal scales that ensured us to locate the center of the SLM coincident with the beam center. We tuned the position and the diameter of diaphragm 2 (A2 in Fig. S1) to let the zero-order light through. The misalignment between the modulated light and the focusing objective (Obj. 1 in Fig. S1) could be corrected by tilting mirror M3 and M4 to ensure the beam normally and concentrically incident Obj. 1 using the image center as the reference. The criterion was to ensure the light center did not move with or without Obj. 1.
3. **Physically correct for the tilt and shift misalignment in the collecting and imaging module.** The misalignment between the two objectives could be corrected using the method proposed at the end of step 2. The difference was that we fine-tuning the X-Y position of imaging objective (Obj. 2 in Fig. S1) in this step, ensuring the beam center was stable with or without Obj. 2. To better observe the situation after the scattering medium, we loaded a flat field pattern on the SLM, and then we fine-tuned M5, tube lens (TL in Fig. S1), and the EMCCD to let the ideal focus locate at the center of the EMCCD chip. We used a multimode fiber (MMF, 25 μm) to collect and direct the light to the PMT and act as the feedback, a 3D translation stage was used to maximize the intensity collected by the MMF measured by the dynamometer. With this setup, the regenerated focus after scattering was supposed to be locate at the same position as the ideal one.

Supplementary Method 2. Recording and calculation of the focal spot size of fCOAT and speckle

We used a camera (EMCCD in Fig. S1) to record the speckle and focus images, to calculate the focal spot size of fCOAT focusing from the images captured, we used interpolation method to calculate the obtained focus. For both small and large focus, the whole focus occupied fewer pixels, so we performed ten-fold cubic interpolation on the images first. Since the regenerated focus were not necessarily symmetrical Gaussian spots, we found the peak value of the focus as the center. A section

line of the intensity profile of the selected image was obtained through the center point, and we chose the half value points between the peak value and the background value on the plotted line as the calculation basis of the FWHM. The scattered light field filled the whole area of the field of view (FOV) of the camera and the size was much larger than that of fCOAT focusing, so we did not calculate the size, but regarded it as a totally scattered light field.

Supplementary Method 3. The adjustments of the system to obtain larger focus

To expand the focus size, we changed the original relay lens (L1=200 mm, L2=200 mm) to another pair (L1 = 300 mm, L2=100 mm) in this experiment. In addition, we reduced the diameter of the iris. Thus the beam was adjusted to 2 mm. According to the optical diffraction limit, $\mathrm{d} = 1.22\lambda/NA$. Before our adjustment, the beam filled the objective lens pupil, which was measured to be 10 mm. The N.A. in such condition was the same as the nominal value of the objective (0.1). After the adjustment, the beam width reduced and the N.A. became smaller and leaded to the focus size became larger. In our experiment, the beam width became one-fifth of the original, thus the focus size became five times. Besides, to better fit the adjusted focus size, we changed the collecting lens pair (the focal lengths of the three lenses after the objective are 30 mm, 75 mm, 30 mm, respectively), so the focus formed by the small N.A. objective could be scaled to fit the collecting area diameter (25 µm).

Supplementary Table 1. Main components used in fCOAT system

Components	Company	Model	Parameters
CW Laser	Changchun New Industries Optoelectronics	MGL-FN-589-500mW	Wavelength: 589 nm<br>Peak power: 500 mW
	Coherent	Sapphire 488-300 CW CDRH	Wavelength: 488 nm<br>Peak power: 300 mW
	MPB Communication	2RU-VFL-500-560-B1R	Wavelength: 560 nm<br>Peak power: 500 mW
Shutter	Daheng Optics	GCI-73<br>GCI-7102M	
Achromatic Doublet	Thorlabs	AC254-030-A	f = 30 mm
		AC254-050-A	f = 50 mm
		AC254-060-A	f = 60 mm
		AC254-080-A	f = 80 mm
		AC254-100-A	f = 100 mm
		AC254-200-A	f = 200 mm
		AC254-300-A	f = 300 mm
Mirror	Thorlabs	BB1-E02	400 - 750 nm
	Union Optic	BDM0025-400	400 - 750 nm
Pinhole	Thorlabs	P25H	25 ± 2 μm
Iris	Thorlabs	SM1D25	Ø1 - Ø25 mm
Polarizing Beamsplitter	Thorlabs	CCM1-PBS251/M	420-680 nm
Half Wave Plate	Thorlabs	WPA2420-450-650	450-650 nm
Beam Block	Thorlabs	LB1/M	400-700 nm
Polarizer	Union Optic	SHP1020	400-700 nm
Spatial Light Modulator	Meadowlark	A512-0532-P8	
Objective	Olympus	PLN4X	4x, NA = 0.1
	Nikon	CFI Plan Apochromat Lambda 20X	20x, NA = 0.75
		CFI Plan Apochromat Lambda 40XC	40x, NA = 0.95
Dichroic Mirror	Edmund	#69-215	550 nm short pass
	Semrock	FF506-Di03	506 nm long pass
	Semrock	FF562-Di03	562 nm long pass
Filter	Semrock	FF01-536/40	536/40 nm
	Edmund	#87-753	615/20 nm
Photomultiplier Tube	Hamamatsu	H7422P-40	Spectral Response:<br>300 - 720 nm
Preamplifier	Stanford Research Systems	SR570	Maximum Bandwidth: 1MHz

Data Acquisition Card	National Instruments	USB-6366	
Tube Lens	Thorlabs	TTL200MP	f = 200 mm<br>ARC: 400 - 1300 nm
EMCCD	Andor	iXon 897 Ultra	512 x 512 pixels<br>16um pixel size

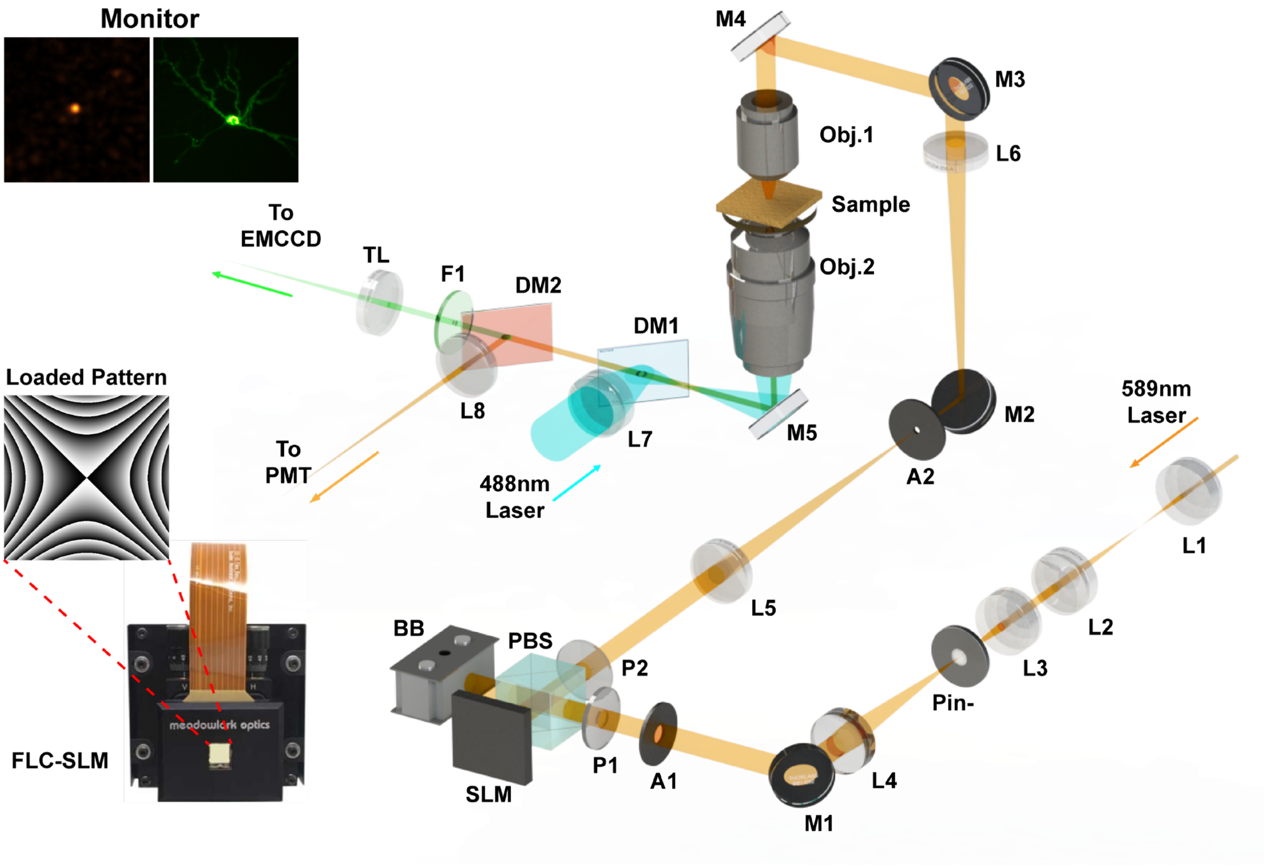

Supplementary Fig. 1. Setup. Schematic of the setup for fCOAT system. L1~L8, achromatic lenses; M1~M4, mirrors; A1&A2, iris; P1, half-wave plate; P2, polarizer; PBS, polarization beam splitter; SLM, spatial light modulator; BB, beam block; Obj, objective lens; DM, dichroic mirror; F, filter; TL, tube lens. The SLM is conjugated to the rear pupil of objective lens 1 to achieve pupil-AO (PAO) modulation. L5 & L6 can be changed to another relay lens pair to reduce the beam diameter to obtain lower focusing N.A. of Obj.1. A pair of lenses can be added after L8 to change the zoom factor of the focus. Filters (F1) can be changed to neutral density filter when recording the focus profile. The loaded astigmatic 'X' image is shown in the left.

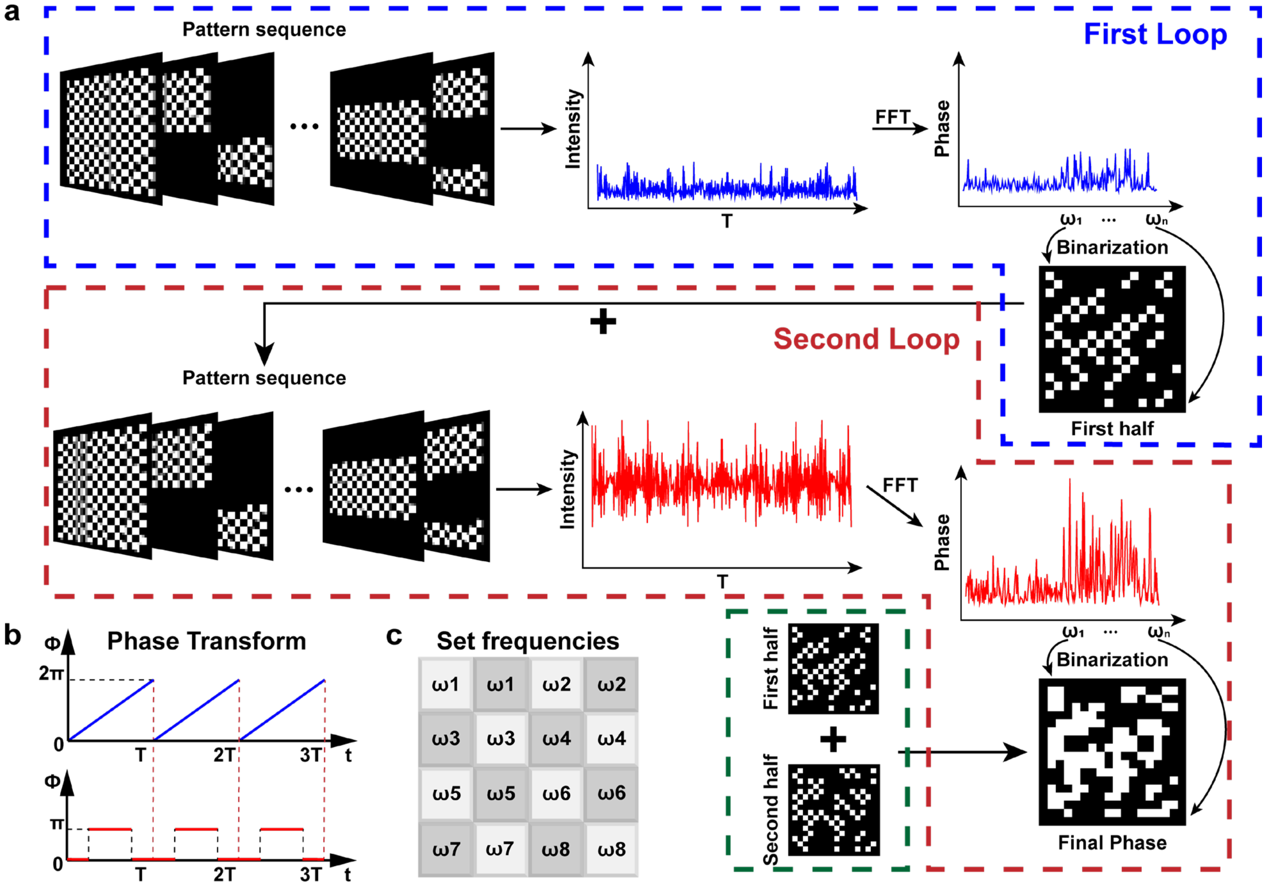

Supplementary Fig. 2. System workflow of fCOAT system. (**a**) Schematics describing the binary coherent optical adaptive technology (fCOAT). (**b**) The transformation principles of loaded phases. (**c**) Frequencies used by different segments during the modulation and collection. The phase patterns are quickly loaded onto the SLM in turn, resulting in a different modulation frequency for each SLM segments. The time-domain signal collected from the target position is transformed to frequency-domain. The phase of each frequency is loaded to the corresponding SLM segments. After the fist loop, half of the total SLM segments are loaded corresponding phase, and then the second loop to determine the phase of the rest segments.

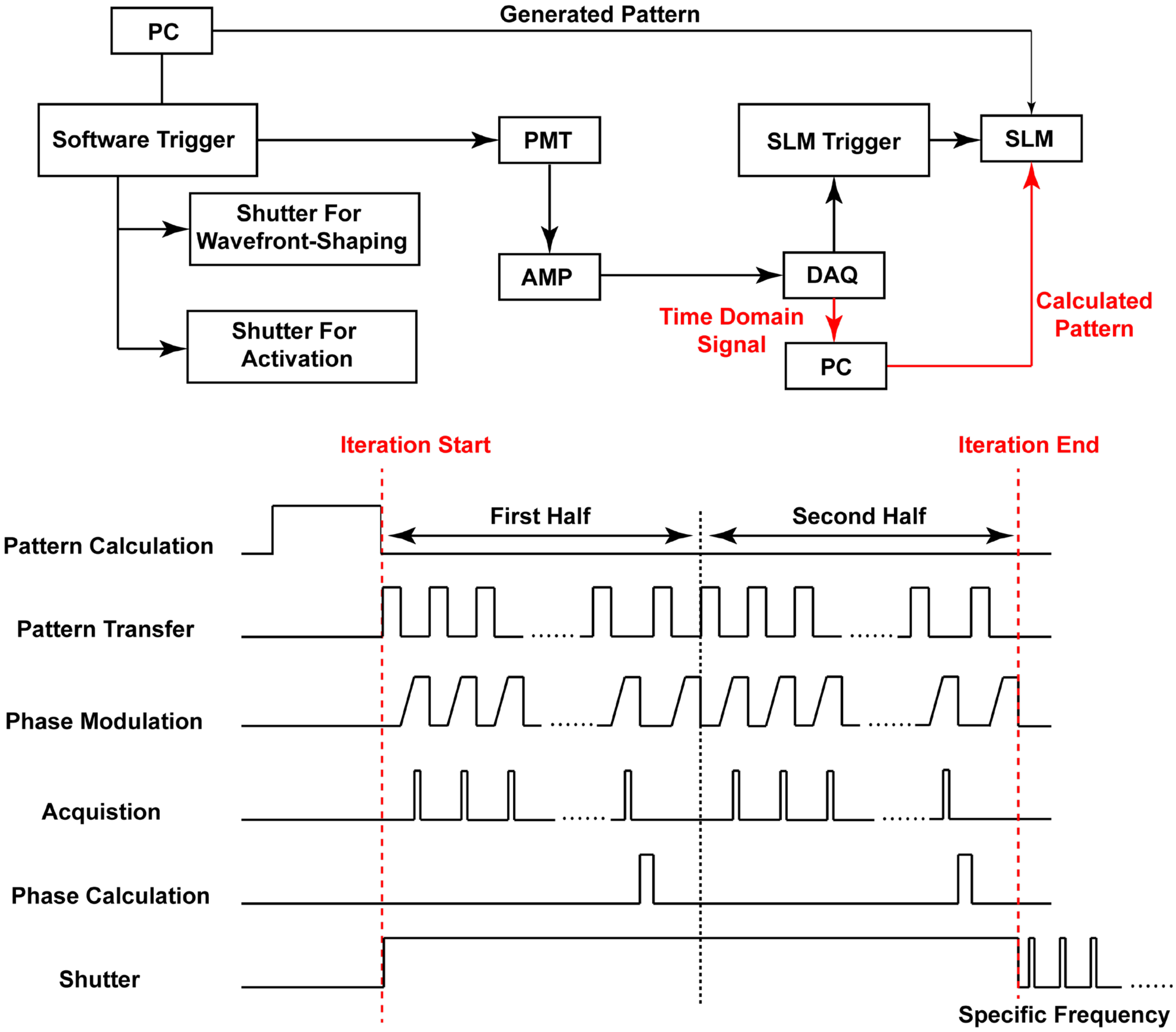

Supplementary Fig. 3. Electrical signal flow diagram. The experiment can be divided into two phases. In the first phase, the fCOAT focusing system gets a correct wavefront solution to overcome the scattering caused by the brain tissue slices. After that, we use the modulated light field to activate the actuator to achieve optogenetics manipulation. To obtain a correct wavefront solution, we implement one iteration of the fCOAT focusing process. During this process, the shutter is open by the control of software. After the focusing process, the shutter is controlled to open at a specific frequency that match the requirements of the photosensitive protein. For each phase pattern, it costs 0.6 ms to transfer the data and another 0.45 ms for liquid crystal cells to response. We set the sample rate of the PMT as 2 MHz, and it takes 400 samples per phase pattern, thus the time for each acquisition is 0.2 ms. To ensure the phase is stable during the acquisition, we set another 0.3 ms for the FLC-SLM as delay. Abbreviations: PMT, photomultiplier tube; AMP, amplifier; DAQ, data acquisition card.

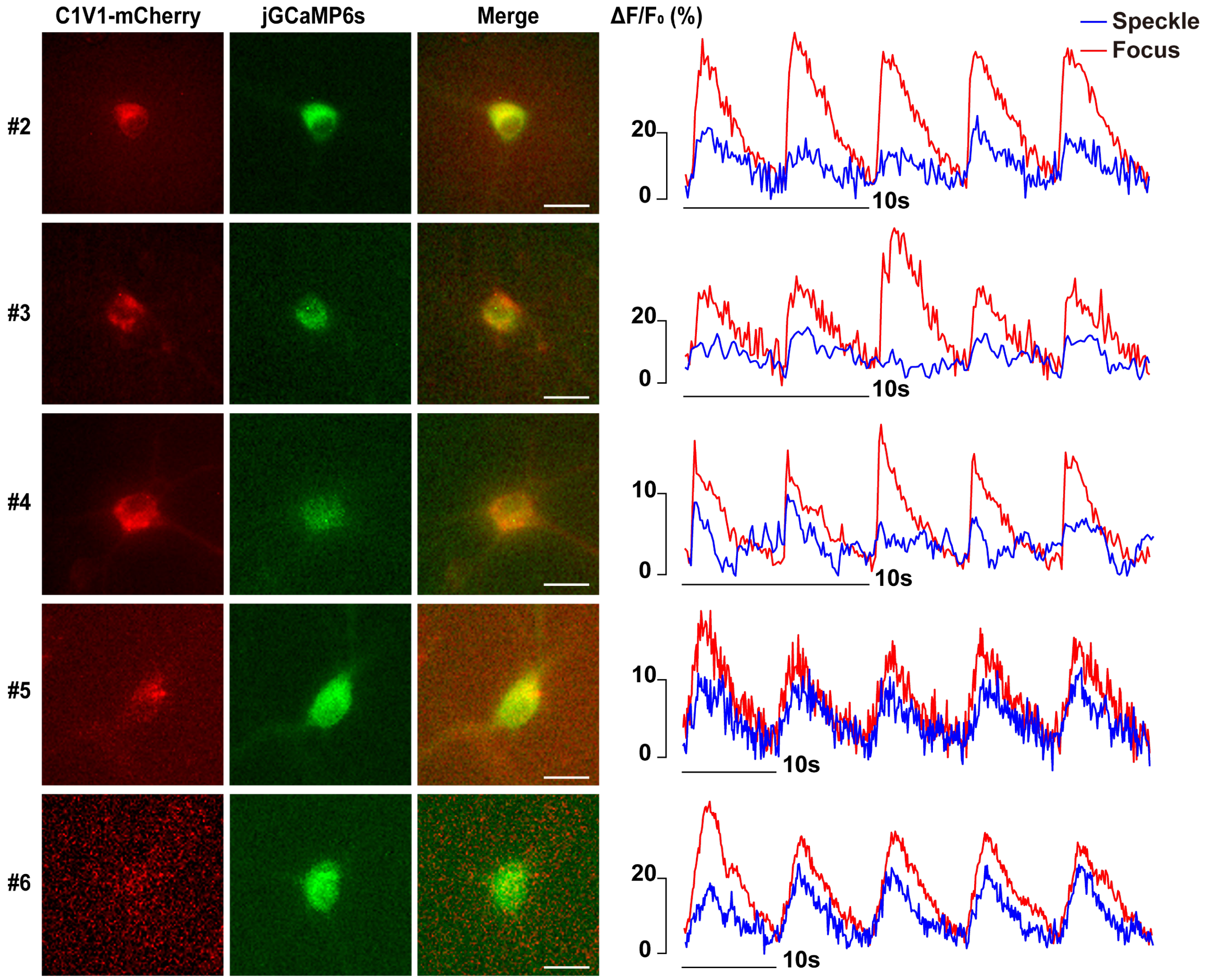

Supplementary Fig. 4. Co-expressed images and calcium transient of neurons. Note that neuron #1 was described in **Fig. 4b, c, d, e**. Different neurons were given different stimulation time sequences. #2: 100 ms stimulation, 5 s period. Comparison of peak neural response (mean ± SE) to different types of stimuli, focus (41.81 ± 1.15% ΔF/F) versus speckle (14.85 ± 1.91% ΔF/F), ****P<0.0001. #3: 100 ms stimulation, 5 s period. focus (31.33 ± 3.50% ΔF/F) versus speckle (10.45 ± 1.18% ΔF/F), ***P<0.0005. #4: 200 ms stimulation, 5 s period. focus (14.94 ± 0.67% ΔF/F) versus speckle (6.72 ± 0.64% ΔF/F), ****P<0.0001. #5: 2 s stimulation, 10 s period. focus (14.45 ± 0.69% ΔF/F) versus speckle (9.27 ± 0.93% ΔF/F), **P<0.0021. # 6: 2 s stimulation, 10 s period, strong (28.62 ± 1.89% ΔF/F) versus weak (18.23 ± 0.97% ΔF/F), **P<0.0021. Scale bar, 20μm.

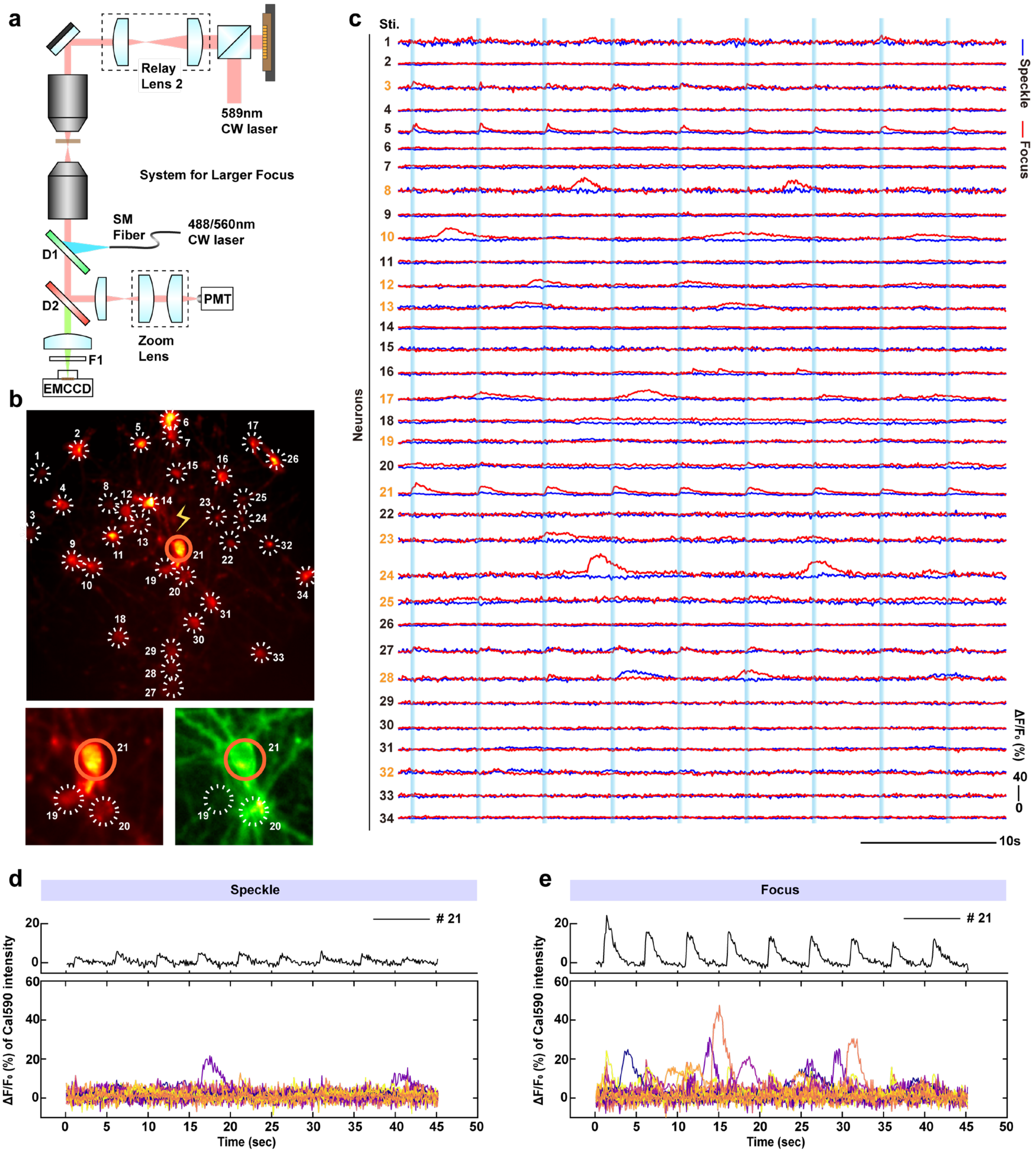

SupplementaryFig. 5. Adjusted system and calcium transient of all neurons. (**a**) Adjusted system setup to obtain larger focus for the experiments. (**b**) Targeted activation of calcium signaling in representative neurons. Red indicates Cal590 fluorescence signal. Green indicates the expression of the ChR2. Neuron 21 is the target stimulation neuron, and all the neurons in this view field are measured. The inset shows the magnification of neurons #19, #20, and #21. (**c**) Individual traces of Cal590 fluorescence in 34 different ChR2 expressing neurons under 488 nm illumination, using extended focusing and speckle light fields. Neurons with at least one effective calcium response are selected in green boxes. The red line is focus, the blue line is speckle. (**d**) (**e**) Traces of Cal590 fluorescence in different ChR2 expressing neurons under 488 nm illumination using speckle and focus light fields. The traces of target neuron are additionally plotted (Top row).

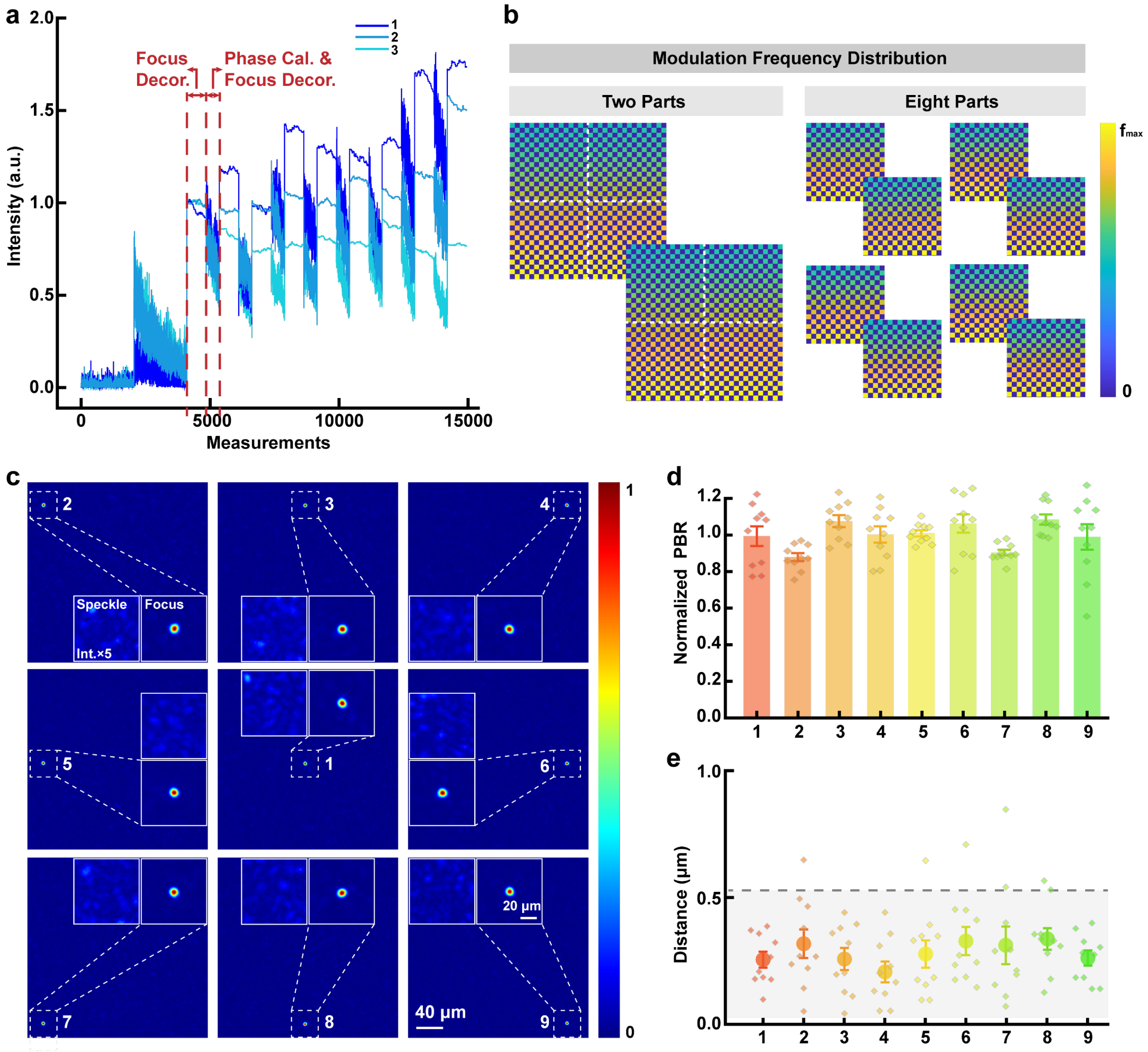

Supplementary Fig. 6. Extra features. (**a**) The intensities of target points during continuous working mode. Each calculation determines only one-eighth of the whole SLM. (**b**) Modulation frequency distribution of each SLM segment in one-time compensation mode (two parts) and continuous compensation mode (eight parts) used to obtain results in (a). (**c**) Focusing at different locations by moving the collection point. The insets show the speckle and focus pattern before and after wavefront compensation, the intensities of speckle patterns are enhanced five times. Scale bars are shown in the figures. (**d**) The statistical data of PBR of focus formed at different locations. The results are normalized using the average value of the PBR of focus of all different locations. (**e**) The statistical data of the distance between focus position and the expected position of different focus locations. Error bars in (d) and (e) represent the SE of ten measurements taken at random different locations on brain slices.

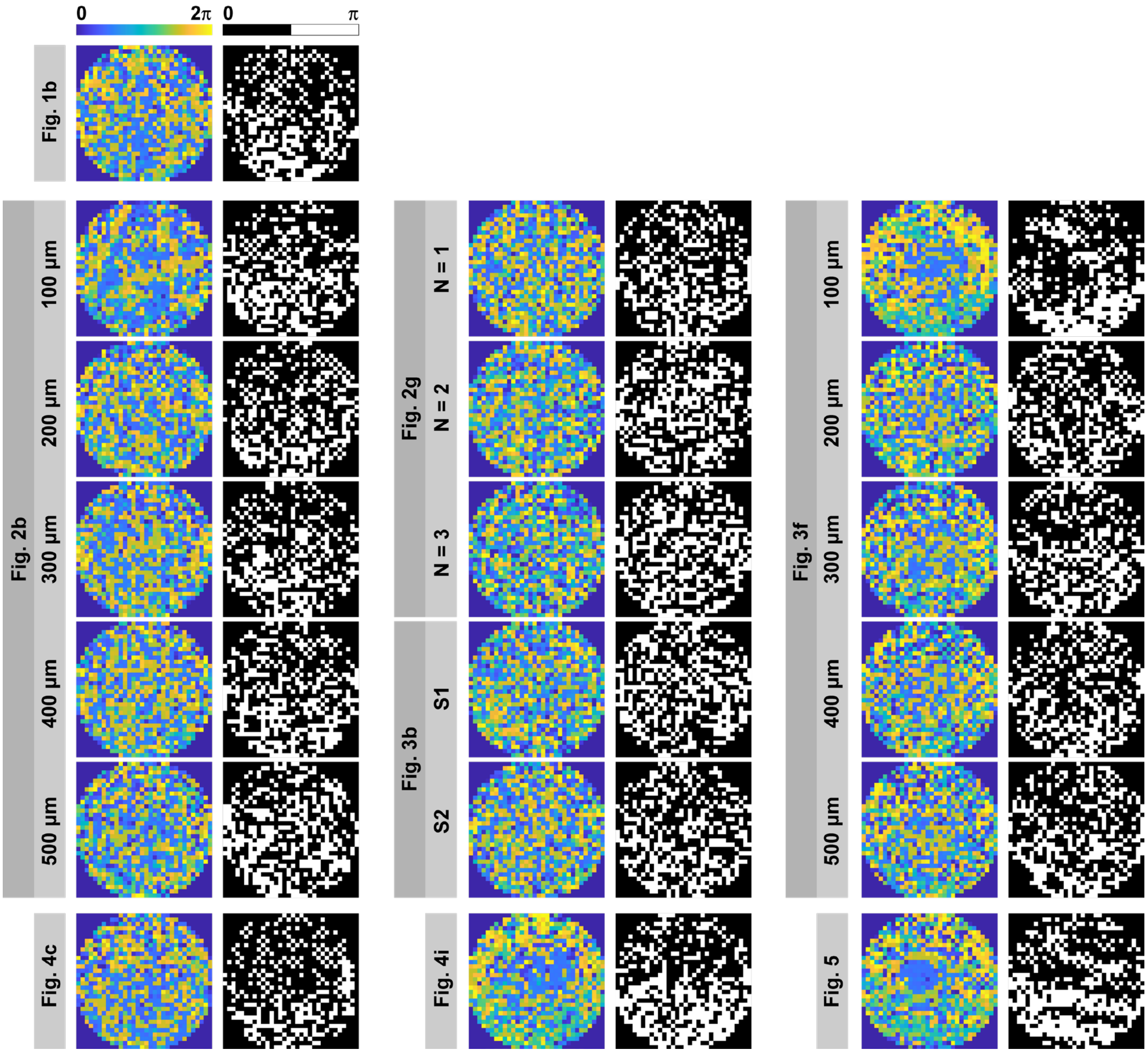

Supplementary Fig. 7. Phase compensation patterns used in focusing figures in the article. The labels are the same as in the article.
