# Supplementary material for: Robust and adjustable dynamic scattering compensation for high-precision deep tissue optogenetics
Source: Commun Biol. 2023 Jan 31;6:128. doi: 10.1038/s42003-023-04487-w (PMC9889738; doi:10.1038/s42003-023-04487-w)
Supplement: Supplementary file 3 — Description of Additional Supplementary Files [file 42003_2023_4487_MOESM3_ESM.pdf]

Description of Additional Supplementary Files

File name: Supplementary Data 1
Description: The source data behind the graphs in the main article.

File name: Supplementary Data 2
Description: The source data behind the graphs in the Supplementary Information.

File name: Supplementary Software 1
Description: The simulation code for fCOAT.
