# Supplementary material for: Robust and adjustable dynamic scattering compensation for high-precision deep tissue optogenetics
Source: Commun Biol. 2023 Jan 31;6:128. doi: 10.1038/s42003-023-04487-w (PMC9889738; doi:10.1038/s42003-023-04487-w)
Supplement: Supplementary file 7 — Reporting Summary [file 42003_2023_4487_MOESM7_ESM.pdf]

nature portfolio

Corresponding author(s): Professor Ke Si
Last updated by author(s): Dec 30, 2022

Reporting Summary

Nature Portfolio wishes to improve the reproducibility of the work that we publish. This form provides structure for consistency and transparency in reporting. For further information on Nature Portfolio policies, see our Editorial Policies and the Editorial Policy Checklist.

Statistics

For all statistical analyses, confirm that the following items are present in the figure legend, table legend, main text, or Methods section.

n/a	Confirmed	
☐	☒	The exact sample size (*n*) for each experimental group/condition, given as a discrete number and unit of measurement
☐	☒	A statement on whether measurements were taken from distinct samples or whether the same sample was measured repeatedly
☐	☒	The statistical test(s) used AND whether they are one- or two-sided<br>*Only common tests should be described solely by name; describe more complex techniques in the Methods section.*
☒	☐	A description of all covariates tested
☒	☐	A description of any assumptions or corrections, such as tests of normality and adjustment for multiple comparisons
☐	☒	A full description of the statistical parameters including central tendency (e.g. means) or other basic estimates (e.g. regression coefficient) AND variation (e.g. standard deviation) or associated estimates of uncertainty (e.g. confidence intervals)
☒	☐	For null hypothesis testing, the test statistic (e.g. *F*, *t*, *r*) with confidence intervals, effect sizes, degrees of freedom and *P* value noted<br>*Give P values as exact values whenever suitable.*
☒	☐	For Bayesian analysis, information on the choice of priors and Markov chain Monte Carlo settings
☒	☐	For hierarchical and complex designs, identification of the appropriate level for tests and full reporting of outcomes
☒	☐	Estimates of effect sizes (e.g. Cohen's *d*, Pearson's *r*), indicating how they were calculated

Our web collection on statistics for biologists contains articles on many of the points above.

Software and code

Policy information about availability of computer code

Data collection	Custom written LabVIEW 14.0 control software was used to perform wavefront shaping and save the wavefront compensation phase patterns used.<br>Andor SOLIS 4.31.30024.0 (iXon) was used to acquire images of the speckle and focus and time stack of neuron images during optogenetics manipulation.
Data analysis	FIJI ImageJ was used for image intensity acquiring (e.g. mean and peak values of focus images, mean values of regions of interests in neuron images to measure fluorescence changes).<br>Custom written Matlab R2019b scripts was used for further signal generating (e.g. line profiles, PBR, and position drifts of focus, signal normalization, calculation of deltaF/F), image background subtraction and interpolation and heatmap plotting. FluoroSNNAP was used for synchronization analyzing of neurons.<br>Origin 2021b, 2022b was used for data plotting (e.g. line plot, bar plot, interval plot).<br>Graphpad Prism 8.0.2 was used to perform a twotailed unpaired t-test.

For manuscripts utilizing custom algorithms or software that are central to the research but not yet described in published literature, software must be made available to editors and reviewers. We strongly encourage code deposition in a community repository (e.g. GitHub). See the Nature Portfolio guidelines for submitting code & software for further information.

Data

Policy information about availability of data

All manuscripts must include a data availability statement. This statement should provide the following information, where applicable:

- Accession codes, unique identifiers, or web links for publicly available datasets
- A description of any restrictions on data availability
- For clinical datasets or third party data, please ensure that the statement adheres to our policy

The main datasets generated and/or analysed during this study are available as source data files.

Human research participants

Policy information about studies involving human research participants and Sex and Gender in Research.

Reporting on sex and gender	No human participants were involved in our study.
Population characteristics	No human participants were involved in our study.
Recruitment	No human participants were involved in our study.
Ethics oversight	No human participants were involved in our study.

Note that full information on the approval of the study protocol must also be provided in the manuscript.

Field-specific reporting

Please select the one below that is the best fit for your research. If you are not sure, read the appropriate sections before making your selection.

☒ Life sciences ☐ Behavioural & social sciences ☐ Ecological, evolutionary & environmental sciences

For a reference copy of the document with all sections, see nature.com/documents/nr-reporting-summary-flat.pdf

Life sciences study design

All studies must disclose on these points even when the disclosure is negative.

Sample size	For focusing data showed in the study, brain slices collected from two mice in total (100 um to 500 um) were used. Each brain slice was divided into four regions (left-front, left-back, right-front and right-back), we performed wavefront shaping for both small and large focus on 2 random positions in one region, which means 8 times focusing processes in total for one brain slice, and 16 times focusing in total for each depth, to ensure similar results are reproducible. For focusing through mouse skulls, we performed 11 times focusing on random positions for each different number of pieces of skulls. For dynamic scattering compensation, the experiments were carried out on a rat ear for 8 times on random positions. Optogenetic stimulations were carried out for more than 80 times across 8 neurons, and over 80 trails of calcium signals were recorded. The experimental procedure and system operation are all standardized in this study. Above all, we believe that the sample size in this study is sufficient for supporting the results.
Data exclusions	We only exclude the data whose quality was compromised by identifiable reasons, such as viral expression failure.
Replication	The results were based on sufficient sample size. We repeatable performed wavefront shaping to form small or large focus down to 500 um deep on 16 random positions on brain slices of each depth , we also performed wavefront shaping to form small focus down to 3 pieces of overlapping mouse skulls deep on 11 random positions. For dynamic scattering study, we repeat the wavefront shaping process on 8 different positions on a same rat ear. For optogenetic study, we repeat the enhanced focusing on 8 neurons for 5 to 10 times stimulation.
Randomization	No randomization was necessary since no group allocation was performed.
Blinding	No blinding was necessary since no group allocation was performed.

Reporting for specific materials, systems and methods

We require information from authors about some types of materials, experimental systems and methods used in many studies. Here, indicate whether each material, system or method listed is relevant to your study. If you are not sure if a list item applies to your research, read the appropriate section before selecting a response.

Materials & experimental systems

n/a	Involved in the study
☒	☐ Antibodies
☒	☐ Eukaryotic cell lines
☒	☐ Palaeontology and archaeology
☐	☒ Animals and other organisms
☒	☐ Clinical data
☒	☐ Dual use research of concern

Methods

n/a	Involved in the study
☒	☐ ChIP-seq
☒	☐ Flow cytometry
☒	☐ MRI-based neuroimaging

Animals and other research organisms

Policy information about studies involving animals; ARRIVE guidelines recommended for reporting animal research, and Sex and Gender in Research

Laboratory animals	Male Sprague-Dawley pups (postnatal day 0, P0) were used to prepare cultured hippocampus neurons; Adult male Sprague-Dawley rats (8-week-old) were used to maintain the expected life activities; P56-70 wild-type C57BL/6J mice were used to prepare brain slices and skulls. All mice or rats used in this study were group housed with a 12/12-h light/dark cycle (lights on at 07:00), controlled temperature (25 ℃) and humidity (60%) and free access to food and water.
Wild animals	No wild animals were used.
Reporting on sex	All the mice or rats used in this study were male.
Field-collected samples	No field-collected samples were used.
Ethics oversight	All mouse related experiments were carried out following protocols approved by the Institutional Animal Care and Use Committees of Zhejiang university of China.

Note that full information on the approval of the study protocol must also be provided in the manuscript.
